# Assessment of fully automated antibody homology modeling protocols in molecular operating environment

**DOI:** 10.1002/prot.24576

**Published:** 2014-04-23

**Authors:** Johannes K X Maier, Paul Labute

**Affiliations:** Chemical Computing Group, Inc.Montreal, Quebec, H3A 2R7, Canada

**Keywords:** homology modeling, comparative modeling, antibody structure, antibody engineering, CDR-H3 loop modeling, loop enrichment, loop refinement

## Abstract

The success of antibody-based drugs has led to an increased demand for predictive computational tools to assist antibody engineering efforts surrounding the six hypervariable loop regions making up the antigen binding site. Accurate computational modeling of isolated protein loop regions can be quite difficult; consequently, modeling an antigen binding site that includes six loops is particularly challenging. In this work, we present a method for automatic modeling of the *F*_V_ region of an immunoglobulin based upon the use of a precompiled antibody x-ray structure database, which serves as a source of framework and hypervariable region structural templates that are grafted together. We applied this method (on common desktop hardware) to the Second Antibody Modeling Assessment (AMA-II) target structures as well as an experimental specialized CDR-H_3_ loop modeling method. The results of the computational structure predictions will be presented and discussed.

## INTRODUCTION

The hallmark feature of antibodies is their ability to bind an almost unlimited collection of target structures with remarkably high affinity. This high target specificity combined with modular composition into distinct functional and structural domains makes immunoglobulins particularly attractive for use as drugs. Antibodies have been successfully applied in therapeutic contexts for the last 20 years and there are currently hundreds of antibody-based drugs in the late stage of clinical trials.^1^ The focus of engineering efforts revolves around the *F*_V_ region of the immunoglobulin, the smallest fragment of the antibody that retains antigen binding ability. One particular challenge in modeling an antigen-binding site is that the regions of interest—the six hypervariable loop regions—are designed by nature to be diverse, whereas the region outside the antigen binding loops, the framework region (FR), is structurally characterized by a conserved beta barrel fold.

Accurate computational modeling of isolated protein loop regions can be quite difficult; consequently, modeling an antigen binding site that includes six loops is particularly challenging. Fortunately, five of the six hypervariable “CDR” loops, three of the light chain (CDR-L_1_, CDR-L_2_, and CDR-L_3_) and two of the heavy chain (CDR-H_1_ and CDR-H_2_) assume a rather small repertoire of main chain conformations and are therefore called “canonical” loops^2^–^4^ leaving the central challenge of modeling the CDR-H_3_ loop. A further complication is the fact that the antigen-binding site has to be modeled as a dimer composed of the variable domains of the light V_L_ and heavy V_H_ chains. Given that the CDR loops bind to the antigen at the interface of the V_L_ and V_H_ chains, their relative orientation also affects binding and can create an additional source of diversity.

A computational assessment of various antibody modeling programs was held in 2009,^5^ the First Antibody Modeling Assessment (AMA-I). Our various submissions in AMA-I were characterized by the amount of user intervention necessary to model the target as well as a control protocol that used no antibody-specific knowledge. Our primary fully automated protocol used in AMA-I, referred to as “autoFv,” has undergone several modifications since its first implementation and is the standard for automated antibody homology modeling in the Molecular Operating Environment^6^ (MOE) software.

In this work, we report on our contributions to the Second Antibody Modeling Assessment (AMA-II) that consisted of two stages: Stage 1 involved full structure modeling (as in the AMA-I) while Stage 2 focused on H_3_ modeling. The autoFv protocol (also referred to as “ccg3” in this work) was used along with other prototype protocols to be described below. Our efforts for AMA-II were directed at rapid automated modeling of antibodies; that is, running on common desktop hardware without user intervention. We describe our methods in the Materials and Methods section. In the next section, the results of AMA-II computational experiments are presented and discussed. Conclusions are drawn in the final section.

## MATERIALS AND METHODS

The MOE antibody modeler is based upon the use of a precompiled antibody structure database, the “Fab Database” (see below). This database is used as a source of framework and hypervariable region structural templates, which are grafted together (see below). Typically, the Fab Database will contain Protein Data Bank^7^,^8^ (PDB) structures augmented with additional proprietary structures, if available. In this work, only PDB structures available as of December 2012 were used, a total of 1,969 structures. Given the V_L_ and V_H_ amino acid sequences as input, the automated antibody modeling protocol (autoFv) proceeds as follows:

1. Search the Fab Database sequences for collections of candidate templates for each of V_L_, V_H_, and the FR *F*_V_ = (V_L_,V_H_) as well as individual CDR loops.
2. Build 10 crude models from combinations of the template candidates. Each model is built by grafting the loop templates onto the template V_L_ and V_H_ framework chains. In case of hetero template composition, the V_L_ and V_H_ grafted templates are transposed via backbone superposition to *F*_V_ dimer coordinates of the highest ranking (see below) *F*_V_ dimer of the V_L_ + V_H_ search results. Side chains are modeled with a rotamer library and coordinates are refined using forcefield energy minimization to relieve strained geometries and clashes.
3. Build a consensus model from the pool of crude models. The model with the lowest *F*_V_ binding energy is used as the consensus structural core.^9^ The binding energy of the *F*_V_ complex is calculated as *E* (V_L_:V_H_) – *E* (V_L_) – *E* (V_H_). Cluster the CDR conformations in the pool and select the best representative (see below) for each CDR loop. Graft the selected loops onto the consensus structural core using the protocol for building the crude models.

**Figure 1:**
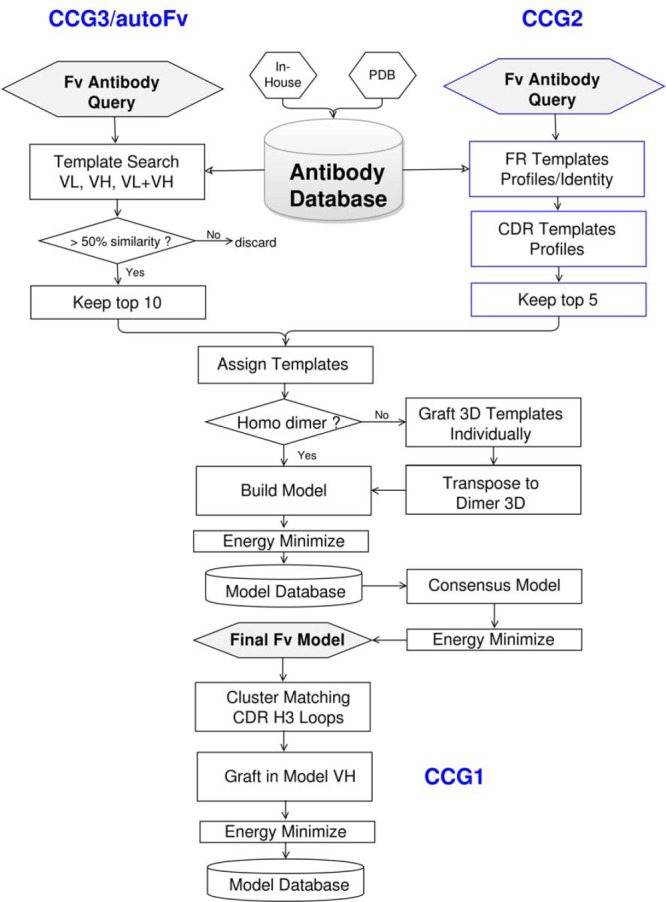
Workflow diagram of the automated antibody homology modeling protocol. The *F*_V_ consensus model is built based on a collection of either 10 (ccg3) or 5 (ccg2) individual models. The steps that are specific to the ccg2 protocol are outlined in dark blue color; ccg1 is an extension step to ccg2 or ccg3 in generating additional loop conformation in CDR-H_3_.

This autoFv procedure is termed “ccg3” for AMA-II; the “ccg1” and “ccg2” prototype protocols are derivatives of ccg3 that will be described after the details of the ccg3/autoFv protocol are described. All three protocols are summarized in the flow chart of Figure 1.

### Fab database compilation

The Fab Database is a collection of antibody structures determined by x-ray crystallography. It was assembled using an automated procedure that identifies *F*_V_ domains from the PDB (or collection of proprietary structures). The antibody detection and classification system is based on a previously published^10^ collection of V_L_ and V_H_ reference sequences enriched with antibody sequences submitted to the PDB with no restrictions concerning type or species. The reference sequences were aligned using MOE, followed by single-linkage clustering to extract antibody clusters of maximum diversity in the framework sequence. The V_L_ reference set is composed of 101 antibody structures with a maximum framework sequence identity of 85% and the V_H_ set of 85 structures at a maximum framework identity of 75%.

The entire PDB was then screened for immunoglobulin structures. The sequence of each protein chain is aligned against the V_L_ and V_H_ reference sets and the sequence identity is calculated as a percentage of identical residues within the aligned *F*_V_ domain as well as within the respective FRs FR_1_, FR_2_, and FR_3_. Protein chains are classified as antibody chains if the overall sequence identity with one chain of the reference collections exceeds 40%. Classifying an *F*_V_ chain as either V_L_ or V_H_ requires a framework sequence identity of at least 60% with one class and <50% with the opposite class. The cutoffs in the antibody detection as described above ensured no contamination of other immunoglobulin-like structures such as T-cell receptor sequences. If detected as a V_L_ chain, further subclassification into either *κ* or *λ* subclasses is achieved by aligning to reference germline sequences originating from the Immunogenetics database.^11^,^12^ For structural clustering, the Structural Composition of Protein database SCOP^13^,^14^ was used as seed clusters followed augmented with single linkage clustering.

### Template search

Given the V_L_ and V_H_ amino acid sequences of the antibody to be modeled, the Fab database is searched for suitable templates. Separate results are collected for V_L_, V_H_, and V_L_ + V_H_ as well as the hypervariable loop regions. A sequence alignment with the BLOSUM62^15^ substitution matrix is performed. Two different scores are used for framework and hypervariable loop regions. The scores for the FRs are calculated as the identity with prealigned framework clusters in the Fab Database, factoring gaps into the calculation. The hypervariable loop scores are calculated as similarity of the query loop compared to the corresponding hypervariable loop sequences of the same length in the Fab Database.^16^ Since the template search is confined to the content of the Fab Database, the time required to find suitable templates takes on average <0.5 s per query. Only the top 10 scoring framework candidates from each of respective result set are retained. If the sequence identity of a V_L_ + V_H_ result is within 10% of the target sequence then a homo template (V_L_ and V_H_ belong to the same structure) is used, otherwise hetero templates (V_L_ and V_H_ from different structures) are considered. For the CDRs, only the top 10 ranking candidates at a minimum sequence similarity of 50% are retained. In case the similarity cutoff in a CDR region is not met, and no templates are found as a result, the loop will be modeled using the general loop building procedure of the MOE Homology Modeler instead of the particular antibody techniques described here.

### Template and model construction

Antibody template structures are built by grafting the framework and hypervariable backbone geometries together. The selected CDR template chains are superimposed in the backbone atoms (N, Cα, C, and O) onto the target framework template within three framework residues on either side of the CDR region followed by tethered energy minimization to relax strained geometries in the transition. In case of a hetero template composition, the templates are transposed via backbone superposition to *F*_V_ dimer coordinates of the highest-ranking *F*_V_ dimer of the V_L_ + V_H_ results of the template search. The MOE general Homology Modeler was used to perform sidechain repacking of CDR loop sidechain rotamers as well as framework residues with van der Waals clashes (>1.5 kcal/mol) with the grafted loop residues. For this modeling exercise, all CDR residues were repacked using the conformations from the MOE rotamer library exclusively.

For the consensus model, single-linkage clustering is applied to determine the conformational spread within the respective CDR loops of the models in the pool built above. The near neighbor cutoff for the CDR conformation clusters is set to 0.4 Å for the canonical loops L_1_, L_2_, L_3_, and H_1_, 0.6 Å for the H_2_ and 1.5 Å for H_3_. The loop candidate with the highest structure score within the most frequent CDR conformation was selected as input for the consensus core structure. The structure score, *S*, is a geometric mean with *S* = (*t*^3^ *g*^2^*ob*)^1/7^ where *t, g, o*, and *b* are values between 0 and 1 as follows; *t* assesses backbone topology, bond lengths, angles etc.; *g* is the Ramachandran phi/psi probability; *o* assesses crystallographic occupancies; *b* assesses temperature factors. Each of *t, g, o*, and *b* were calibrated by setting 0 to correspond to the lower range of outliers and 1 the upper range of outliers as measured from PDB statistics. Values of *S* are interpreted as 0 meaning completely unsuitable for modeling purposes and 1 meaning ideal. Only structures with *S* > 0.65 were used in this work.

For the canonical CDR loops, the loop conformations of the crude models are used as input for conformational analysis in each CDR loop category, recruiting the most common conformation as input for the consensus model. The conformational analysis helps filter CDR loop conformations that fit particularly well within a distinct modeling environment, shifting the selection process away from general sequence criteria to structurally more specific ones. Consequently, CDR loops with lower sequence identity (but higher structure scores) may be selected for assembly in the consensus model. In addition, the crude models serve as a pool to address complex structural issues such as the V_L_:V_H_ dimer orientation and the variability in CDR loop conformations that are challenging to predict based on sequence alone.

### Protocol ccg2

The ccg2 protocol is a variation of the autoFv/ccg3 protocol described above. First, only five crude models are built (instead of the usual 10), which reduces the overall modeling time significantly. Second, the canonical CDR loops templates are selected based on position-based profile scores of the respective CDR loop clusters in a special canonical loop database (see below) that match the length of the CDR query; only the top five most sequence-similar candidates are retained. For H3, only the top five template candidates based on sequence similarity calculations are retained.

The canonical CDR loop templates of the ccg2 protocol are selected from a dedicated loop database. All canonical CDR loops extracted from the Fab Database were grouped by CDR type and loop length. Templates with structural issues in the protein backbone and/or low phi/psi scores were discarded. The remainder were clustered based on backbone RMSDs at a resolution of 0.3 Å employing a single-linkage clustering algorithm followed by a sequence profile calculation that determined the frequencies of unique residues in each cluster column. The resulting values were normalized and used as weights for compiling a position score for each residue in the cluster and used when matching the template queries to the sequence profiles of each cluster. A similar approach using Hidden Markov models has been previously reported.^17^ In this way, residues that are more preserved (and thus more relevant for determining the actual conformation) within a cluster have a stronger impact on the selection.

The selection of framework candidates is based on position-based profile scores with residues shown to be involved in *F*_V_ dimer interactions. The orientation between V_L_ and V_H_ is a particular challenge in modeling an antigen-binding site.^18^,^19^ Narayanan *et al*. (2009) used the Cα RMSD in the FR of V_L_ when superposed on the V_H_ framework as a metric to quantify the relative changes in the V_L_:V_H_ orientation and also demonstrated that the native V_L_:V_H_ orientations correspond with the energy minima measured as *F*_V_ complex interaction energies. We make use of this observation along with the residues identified by Abhinandan and Martin^20^ in the framework (L38, L40, L41, L44, L46, L87, H33, H42, H45, H60, H62, H91, H105, and Chothia numbering) for the profile scores. With this distinct and reduced set of residues in place that are (intended to be) determinants of the *F*_V_ dimer orientation, the framework template candidates were identified based on the number of identical residues within this distinct set between the templates. The overall framework sequence identity was used as a secondary metric to determine the final FR template selection.

### Protocol ccg1

The ccg1 is a derivative of the ccg2 protocol in which the CDR-H_3_ loop diversification step is applied using the Fab Database described above. The database was first searched for H_3_ loops identical in length with the query CDR-H_3_. Single-linkage clustering was applied based on backbone RMSD at a resolution of 0.5 Å to determine distinct sets of CDR-H_3_ loop conformations. The candidate with the best structure score within each conformation cluster was used as input for template assembly into the V_H_ chain of the consensus model or native structure. A model based on each cluster was built in the manner described above. For each resulting model, V_L_:V_H_ binding energies and conformational energies were calculated. Models with more than 10 clashes > 0.5 kcal/mol or positive conformation energies were discarded. The three models with the lowest binding energies were output.

### Manual modeling of target MA2-1 in AMA-II Stage 1

The lack of suitable structure templates for CDR L_1_, L_3_, and H_3_ combined with low sequence identity in the FR across species precluded a fully automated modeling approach for target MA2-1. Instead, this target was modeled using the interactive MOE Antibody Modeler application using the default settings. This application suggested PDB:2VUO—one of the few rabbit immunoglobulins in the PDB—as the only suitable structure template. The top 3 ranking models based on geometry assessments in the protein backbone and overall energy scores of 100 coarse models were used for CDR conformation diversification in CDR-L_1_, CDR-L_3_, and CDR-H_3_ using LowModeMD.^21^ The backbone atoms N, Cα, C, and O of the CDR loops L_2_, H_1_, and H_2_ as well as the framework atoms were kept fixed, allowing only the sidechain atoms to move and thus accommodating novel conformations. The procedure was stopped after 300 distinct conformations were generated. The candidates with the lowest energies were selected and subjected to a final structure refinement procedure as described above.

### CDR-H_3_ modeling in AMA-II Stage 2

Stage 2 focused on modeling CDR-H_3_ when given x-ray crystal structures (with H_3_ removed) provided by the organizers of the modeling assessment. Our efforts involved the use of a dedicated CDR-H_3_ loop database. This database was searched to find H_3_ loops (of matching length) that superposed to an RMSD of 0.25 Å to the surrounding three framework backbone atoms. Sidechains were repacked as described above and the coordinates were refined using a tethered protocol. During refinement, all heavy atoms of the input x-ray structure were held fixed allowing only the H_3_ loop atoms to move within the antigen-binding complex. The minimization procedure was repeated five times, gradually releasing the tethers on the backbone atoms of H_3_. Finally, models with unresolvable clashing energies >2.5 kcal/mol were discarded and the best scoring (see below) conformation was selected. On average, the processing time of one conformation consumed ∼1 CPU minute.

For the compilation of the CDR-H_3_ loop database, all H_3_ loops were extracted from the Fab Database and clustered according to length and backbone RMSD. Single-linkage clustering applied based on a backbone RMSD cutoff of 0.4 Å to determine distinct sets of CDR-H_3_ loop conformations. Structures with positive forcefield energies, poor (<85%) Ramachandran map probability, or a clash count of >5 were discarded. The cluster member with the highest score (see below) was used as the representative. The antibody dimer structures of these CDR-H_3_ representatives were then extracted from the Fab Database for further diversification of the loop conformation space by subjecting them to conformational search using LowModeMD. All residue atoms beyond a perimeter of 5.5 Å of CDR-H_3_ loop atoms were kept fixed, as well as the backbone atoms of non-H_3_ residues within the perimeter. This left the H_3_ loop atoms, including three flanking framework residues and all sidechain atoms within the perimeter, free. LowModeMD was terminated after 100 attempts to generate a novel conformation up to a maximum or when 50 unique structures were generated. The structural scores of the newly generated H_3_ conformations were calculated discarding conformations with inappropriate structure/clashing scores. The remainders were clustered at a resolution of 0.4 Å and the conformation representatives were merged with the corresponding conformation pool extracted from the Fab Database.

As a control alternative to energy-based scores used for ranking output models in Stage 1, a scoring function was developed factoring in H_3_-specific properties like accessible surface area, psi/phi values and interaction energies of CDR-H_3_ to the *F*_V_ dimer complex. The prepared representative *F*_V_ dimer structures extracted above from the Fab Database were used as raw data. For each loop length category (ranging from 4 to 14 residues), the respective components were calculated, normalized, and the frequency data was fitted to a Gaussian distribution. The final CDR-H_3_ loop score was calculated as the geometric mean of the three individual loop scores.

### Hardware and software

All calculations (and validation analyses) in this work were conducted using the MOE version 2012.10 on two computers, a Mac Pro, Dual Core Intel Xeon at 2.66 GHz, OS X 10.5 (Leopard) and a Dell Inspiron, Intel 2 Dual Core on OS Linux Mint 13. Energy calculations and coordinate refinement were performed using the AMBER 12^22^ forcefield parameters augmented with the GB/VI implicit solvent model^23^ with a nonbonded cutoff of 8 Å. PDB structures were prepared for simulation work using the MOE Structure Preparation application followed by Protonate3D.^24^

## RESULTS AND DISCUSSION

### AMA-II Stage 1

The Stage 1 models (ccg1, ccg2, and ccg3) were built using the methods described in the previous section with the exception of target MA2-1, a rabbit antibody, which was modeled manually due to a lack of templates in the PDB. Each final ccg3 model required 20–50 CPU minutes to compute and 10–25 CPU minutes per final model for ccg2 and ccg3 protocols with most of the time spend in coordinate refinement. All of the protocols presented here produced models of good structural quality evidenced by an overall MolProbity score average of 1.5, lower than the 1.8 average we have calculated for 1,969 x-ray structures from the PDB (our Fab Database). A detailed listing of the various MolProbity measurement components and comparisons to the other participants are provided in the Supporting Information Tables of Topalev *et al*. in this issue.

Table 1 presents a comparison of the ccg1–3 structures with the (previously unknown) x-ray coordinates of the targets of the second antibody modeling assessment. The RMSD values measured over the entire framework of all 10 models (FR) reveals a more robust and overall slightly better performance of the established ccg3/autoFv protocol (0.80 ± 0.19 Å RMSD), compared with ccg2 (0.87 ± 0.24 Å RMSD). However, the discrepancy is largely caused by a poor ccg2 template choice for modeling target MA2-3 with a RMSD of 1.35 Å compared with 0.57 Å of the corresponding ccg3 model. The low agreement in dimer orientation is further evidenced by large tilt angles (14.3°) and large discrepancies in the HL torsion and HC_2_ bend angles (7.8° and 7.9° difference to the x-ray structure). Our analysis revealed that the failure in selecting an appropriate framework template for this target was not solely rooted in a failure of the FR scoring function that was applied in selecting the five template candidates to build the crude models, but rather in the unusual, “open” conformation of CDR-H_3_ in one of the models. Unfortunately, this unusual conformation also measured the lowest *F*_V_ complex binding energy among the five crude models that is the driving parameter for the framework selection of the consensus model. Further analysis of the MolProbity scores (Topalev *et al*. in this issue) and the all-atom energies of the ccg2 model are reasonable for all of the measured components suggest that lower interaction energies were not obtained at the expense of unrealistic structures. It is possible that a larger collection of crude models as used by ccg3/autoFv (ten instead of five) offers better protection against selecting a particularly poor framework template. However, given our findings that CDR-H_3_ residues are strongly influential in the interaction between V_L_ and V_H_ chains, the issues might be more complex and point potentially to a chicken and egg problem by which the orientation of the V_L_ and V_H_ chains may only be predicted accurately if the correct conformation of CDR-H_3_ is known. Even with inclusion of the poor modeling result of target MA2-3, the measurements in the FR are promising and comparable with the other modeling approaches presented in this contest (see Almagro overview in this issue). As the ccg2 modeling protocol was still under development at the time of AMA-II, we are optimistic that further fine-tuning of the scoring parameters will produce more stable results that are in keeping with those demonstrated with ccg3/autoFv over the last few years. The even more experimental ccg1 protocol fared worst of the three. Since ccg1 used the output of ccg2 it would appear that the additional steps were detrimental and further investigation is required to diagnose the cause. An interesting possibility is ccg1's additional coordinate refinement with CDR-H_3_ backbone atoms fixed (letting the other CDR loops adapt to the backbone of CDR-H_3_), which may introduce negative effects on the other CDR loops (e.g., CDR-L_1_ in MA2-2, MA2-3 and MA2-10 and to a lesser degree in CDR-L3 in MA2-3).

**Table I:**
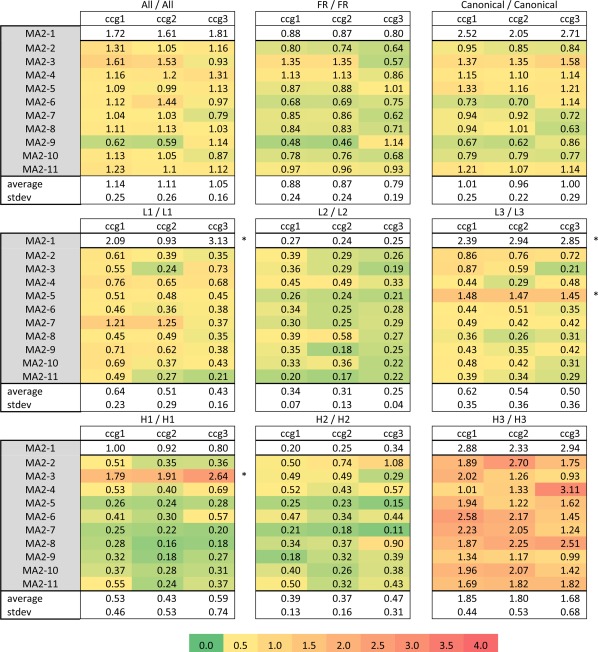
RMSD Values in Å Between Three CCG Protocols (See Text) and the Stage 1 Target X-Ray Crystal Coordinates; “All” is the RMSD on the Entire *F*_V_ Region; “FR” Is the RMSD on the Framework (Excluding the CDR Loops); “Canonical” Is the RMSD on CDR *L*_1_–*L*_3_ and *H*_1_–*H*_2_; “*L*_1_–*L*_3_” and “*H*_1_–*H*_3_” Are the RMSDs of Each Individual CDR Loop Region

All RMSD measurements (in Å) are based on backbone atoms N, Cα, C, and O.

Since the distinct arrangement of CDR loops is a critical factor in the formation of the antigen/antibody complex, CDR measurements must be assessed on two levels–the first with respect to their orientation within the dimer and the second with respect to the accuracy of the individual loop conformations. Assessment is compromised, however, when the entire antigen-binding surface (all Loops) is considered due to the influence of the CDR-H_3_ loop component. Accordingly, the canonical loops are assessed first (Table 1, Canonical) and, in this measurement component, these show competitive results with an average ccg3/autoFv of 1.00 ± 0.29 Å RMSD for all 10 models. The RMSDs are almost identical between the protocols (∼1.0 ± 0.25 Å) with some interesting variations in specific cases. For example, larger RMSD outliers of single models are responsible for the variations in the light chain averages (CDR-L_1_ in MA2-7, 1.25 vs. 0.37 Å and 0.58 vs. 0.27 Å in MA2-8 for CDR-L_2_), whereas overall improvement in modeling CDR-H_1_ and CDR-H_2_ is observed with ccg2 over ccg3. As expected, the less canonical and particularly challenging CDR loops (CDR-L_1_ and CDR-L_3_ of target MA2-1, CDR-H_1_ of MA2-3 and CDR-L_3_ of MA2-5) show lower precision but within the range of the results in the modeling competition (see Table 1 and Teplyakov and Almagro this issue). Promising results are seen in CDR loops with low agreements to the North CDR clustering scheme such as the CDR-L_3_ loops of MA2-2 and MA2-3, CDR-L_1_ of MA2-4 and MA2-5 and CDR-H_2_ of MA2-6 and MA2-8 where the RMSD values are within the modeling precision of less challenging cases. An exception is CDR-H_2_ of MA2-8 that has only been well modeled with the ccg2 protocol (0.35 vs. 0.90 Å RMSD). As mentioned in Teplyakov and Almagro (this issue), improvements could be made in addressing the *cis/trans* configuration of certain proline residues in the CDRs in more detail.

**Figure 2:**
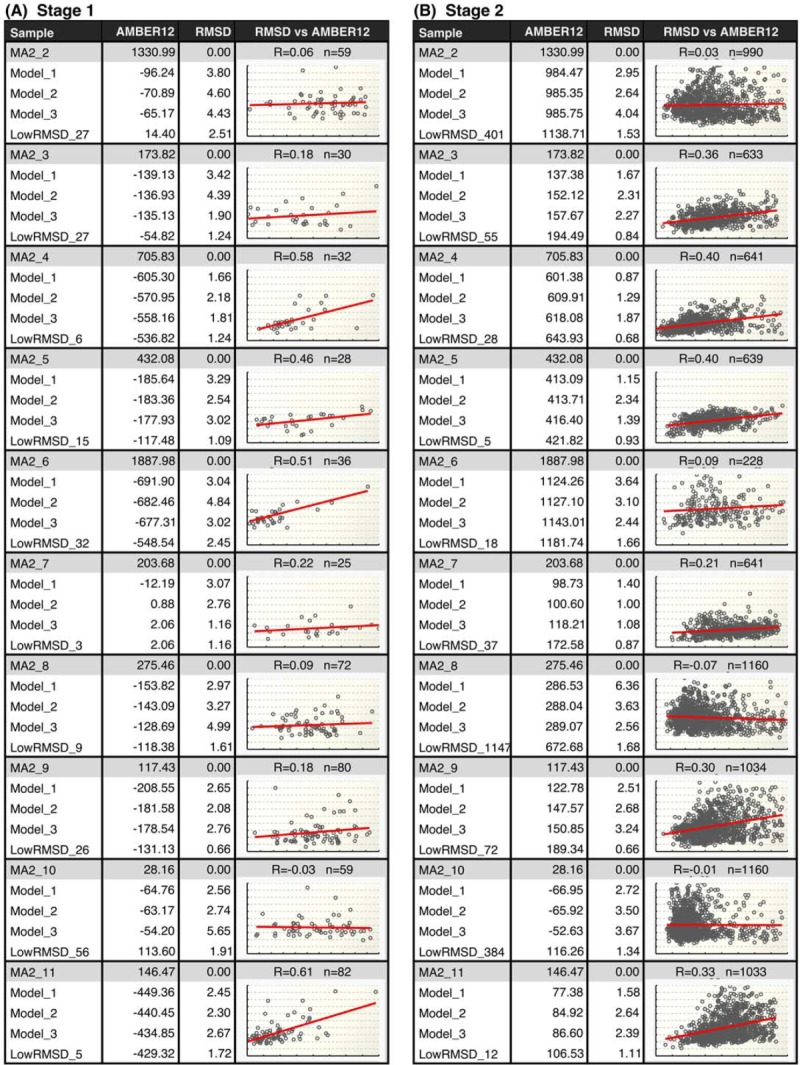
Comparison of AMBER12 all-atom energies (kcal/mol) of the target x-ray crystal structures with the Stage-1 (A) and Stage-2 models (B). Values referring to the X-ray structures are shown with gray background, followed by the lowest three energy models (Model-1, Model-2, and Model-3) and the model with the lowest global RMSD (LowRMSD_x). The suffix indicates the rank in the energy-sorted conformation pool. The RMSD values (Å) are based on measurements in the backbone (N, Cα, C, and O) of CDR H3 and represent the global RMSD to the target H3. The Plots show the results of a correlation analysis (global RMSD against AMBER12 all-atom energies), “*n*” denotes the number of conformations left in the conformation pool after removal of models with bad energies or unresolvable clashes, “*R*” is the Pearson's correlation coefficient of global RMSD (*y*-axis) and Amber12 all-atom energies (*x*-axis).

A quality assessment of the antigen-binding surface is not complete without the analysis of the key CDR-H_3_. The unusually broad range in loop lengths, the conformational flexibility as well as its influence on other areas of the antigen-binding complex warrants a separate evaluation. Since it is not yet possible to employ any type of loop conformation dictionary in modeling this loop accurately, it is probably best to offer a set of plausible conformations as a representation of CDR-H_3_ rather than a single conformation providing only the sequence as input. To this end, the main objective of the ccg1 protocol is to provide a diverse, structurally sound, and energetically plausible collection of CDR-H_3_ conformations. On average, 55 alternate and diverse conformations were generated in approximately one CPU hour of computation on a common desktop computer. While this approach demonstrates that the primary objective was satisfied, the H_3_ modeling results in Table 2 reveal that it was not possible to improve the modeling predictions of CDR-H_3_ compared with the output conformations of ccg2 and ccg3 (which performed best). The ccg1 conformation diversification step does not fail in producing reasonably accurate conformations (average global RMSD of 1.54 ± 0.61 Å) but rather in selecting the best, or close to the best, solution within the generated conformation pool. The H_3_ scoring function, trained on loop-specific properties like psi/phi torsion values, *F*_V_ interaction energies and surface area was implemented as a relative metric for ranking CDR-H_3_ loop conformations but failed in predicting CDR-H_3_ conformations accurately (3.31 ± 0.94 Å). By default, the ccg1 protocol uses energies as a ranking criterion for the models in addition to the loop score. Selecting the lowest energy model for predicting H_3_ would have improved the average RMSD value to 2.89 ± 0.57 Å (and thus within the assessment) but would have also failed to improve the prediction accuracy of CDR-H_3_ of the input models ccg2 or ccg3/autoFv. Figure 2(A) lists the AMBER12 energies of the target x-ray structures and the global RMSDs in CDR H3 of the top 3 ranking ccg1 models (Models 1, 2, and 3) followed by the lowest RMSD model. The suffix indicates the position of the low RMSD conformer within the energy-ranked conformation set. The comparison of AMBER12 all-atom energies shows that the x-ray structures would have been discarded in all cases if present in the pool of ccg1 conformations. The correlation analysis [global RMSD (y-Axis) vs. AMBER12 all-atom energies (x-Axis)] as depicted in the plots of Figure 2(A) does not support the notion that prediction accuracy is linked to low energies of the models further substantiating our problems in discriminating the best or close to the best possible solutions. Taken together, these results suggest that modeling of CDR-H_3_ still represents a serious challenge both for us as well as for the participants that use more computationally expensive methods for modeling H_3_.

**Table II:**
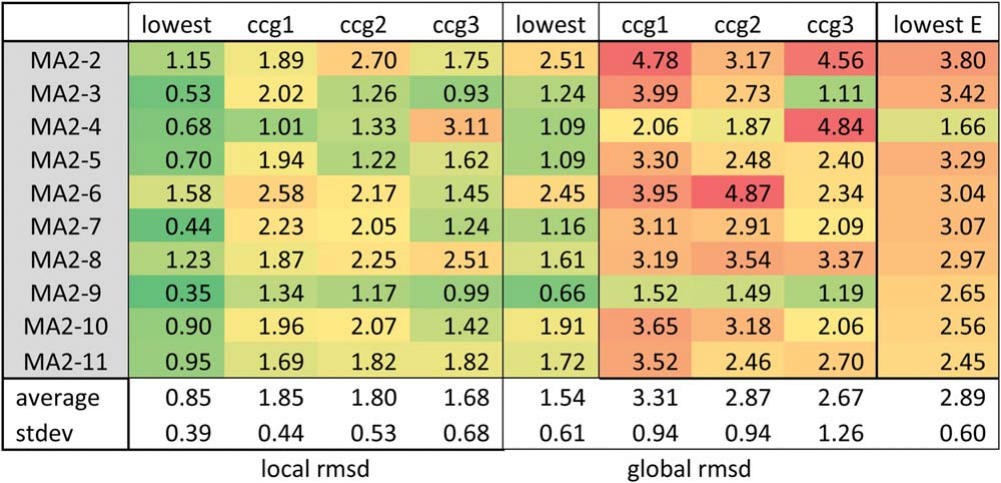
RMSD Values in Å (Local and Global) of H_3_ Between Three CCG Protocols (See Text) and the Stage 1 Target X-Ray Crystal Coordinates; “Lowest” Is the Smallest RMSD Value of Intermediate Models Generated in the Protocols; “ccg1–3” Are the RSMD Values of the Three Protocols; “Lowest E” Is the RMSD of the Intermediate Structure with the Lowest Forcefield Energy for ccg1

Global RMSDs denote loop measurements after superposition of the framework first.

In summary, the fully automated modeling protocols ccg3/autoFv and ccg2 show good prediction accuracy in the structural core and the canonical CDR loop regions. In AMA-II, the more seasoned and established ccg3/autoFv modeling method displayed a more robust and slightly improved performance compared with ccg2 (overall RMSD average of 1.05 vs. 1.11 Å for ccg2 and 1.14 Å for ccg1 Table 1) in contrast to an internal benchmark (data not shown). The variations we observe among these modeling procedures are more evident in the framework/dimer orientation category (see Table 1 FR section as well as Topalev *et al*. and the Supporting Information Section of Almagro *et al*. in this issue) and are signs of a not yet fully fine-tuned and matured ccg2 modeling protocol.

### Comparison to the first antibody modeling assessment (AMA-I)

**Figure 3:**
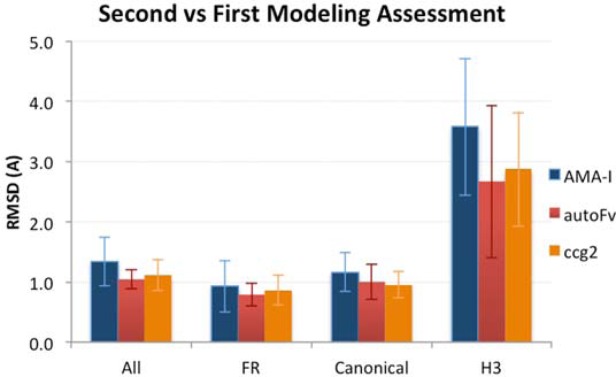
Comparison of the RMSDs of the automated protocols in the first and second antibody modeling assessment in the *F*_V_ region (All), the dimer framework (FR), Canonical Loops (Canonical), and CDR-H_3_ (H3).

The second assessment (AMA-II) provides an opportunity to measure the improvements made since the first assessment (AMA-I). The comparison is best done with the autoFv/ccg3 protocol since this protocol was also used in the first modeling assessment. As depicted in Figure 3, the current version of the autoFv protocol suggests enhancements in the important framework (FR), canonical loops (Canonical) measurements evidenced by the average RMSD values of 1.34 vs. 1.05Å over the entire *F*_V_, 0.93 vs. 0.79 Å in the framework and 1.17 vs. 1.0 Å in the canonical loop categories. A similar degree of progress is seen with the ccg2 protocol. As discussed above, the differences observed in modeling the H_3_ loop are not rooted in a systematic effort to improve modeling in this category but rather a result of refinements made in other areas of the binding site that result in better predictions of CDR-H_3_ overall.

### AMA-II Stage 2

Stage 2 focused on modeling of CDR-H_3_ when given the coordinates of the remaining antibody dimer. Minor deviations in modeling the framework and canonical CDR loops will influence the architecture of the antigen binding surface and will in turn affect the modeling of CDR-H_3_. In this respect, Stage 2 can be viewed as a reduction of the modeling complexity by eliminating the uncertainty of modeling the remaining *F*_V_ region; in other words, Stage 2 is a CDR-H_3_ modeling exercise under ideal conditions. Using the dedicated CDR-H_3_ loop database and techniques described in the previous section (still under development at the time of AMA-II), we generated five models, CCG1–CCG5, selected on the basis of three different scoring regimes. The CCG1 and CCG2 models were the two lowest forcefield energy structures; CCG3 and CCG4 models represent the top two ranking models based on specialized H_3_ loop scores; CCG5 models were selected manually (based on personal experience, atom clashes and protein geometry) from the pool of the top ten candidates of each group.

Table 3 presents the RMSD comparison (local and global) of each of the generated models with the experimental crystal structure. The number of CDR-H_3_ conformations generated was ∼800 conformations for CDR-H_3_ lengths of 8, ∼1,200 for lengths 10 and 11, and ∼300 conformations for the 14 residue loop of target MA2-6. Conformation coverage was good with 7 of 10 targets having a conformation generated at <1.0 Å local RMSD to the experimental structure, two between 1.0 and 1.25 Å and target MA2-6 at 1.6 Å. It appears the conformation space of target MA2-6 was not adequately sampled suggesting that the iteration limits of LowModeMD should increase as a function of loop length. The energy-based selections, CCG1 and CCG2, were better than the scoring function selections, CCG3 and CCG4; this is true on average and in the individual cases with the exception of target MA2-3. In three cases, the manual selection, CCG5, outperformed the CCG1 energy based selections; however, in two out of the three the RMSD to the crystal structure was >2.0 Å and these cases (MA2-6 and MA2-8) also had no generated conformations under 1.0 Å RMSD. This suggests that in the absence of a close-to-experiment conformation, aesthetic qualities of the loop conformation, rather than energy, may lead to closer predictions. The average CCG1 RMSD to experiment on all 10 targets was 1.5 local and 2.5 Å global. Leaving out the targets for which no conformation <1 Å RMSD (local) was generated (MA2-2, MA2-6, and MA2-8) the averages were 1.1 Å RMSD local and 1.7 Å global. This supports the hypothesis that if there is a close conformation to experiment in the pool then the energy based selection can do well.

**Table III:**
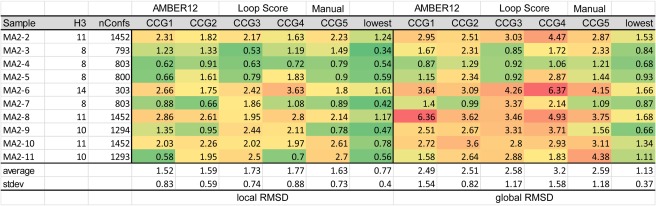
RMSD Values in Å (Local and Global) Between Three Distinct Selection Methods (See Text) Employed in Stage 2 and the Target X-Ray Crystal Coordinates; “H3” Gives the Length of the CDR-H_3_ Loop; “nConfs” Is the Total Number of Conformations Generated in the Respective Pool; “CCG1 and 2” Are the RMSDs of the Lowest AMBER12 All-Atom Energy Models, “CCG3 and 4” Are the RMSDs of the Models With the Highest Loop Scores and CCG5 Is Based on a Manual Selection, “Lowest” Is the Lowest RMSD Conformer in the Pool

All RMSD measurements are based the backbone atoms N, Cα, C, and O.

**Figure 4:**
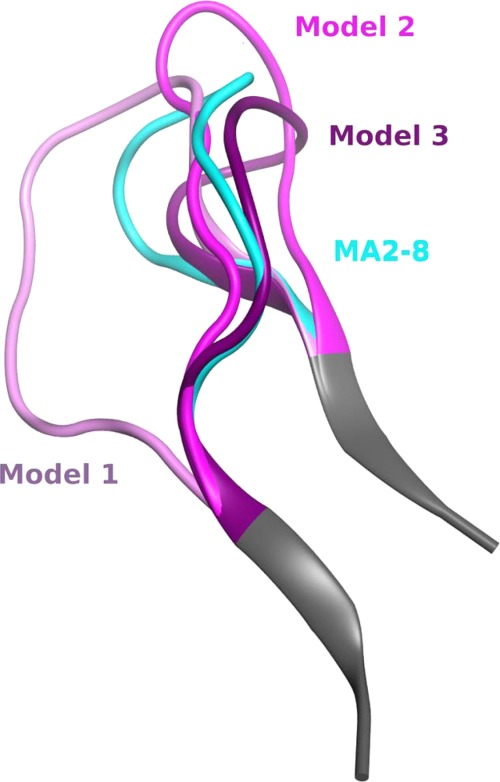
Conformational range in CDR-H_3_ within a small energy window of 2.6 kcal/mol. The energies of Models 1, 2, and 3 are in order from lowest to highest and the deviation of the models from the *x*-ray coordinates were 6.37, 3.63, and 2.56 Å global RMSD.

Some discussion is warranted surrounding the issues related to energy based model selection. First, atomic forcefields are only approximations and, as such, they have parameterization errors; for example, the bonded parameters, van der Waals and electrostatics/solvation treatment. The lowest energy structures can be quite different from each other. For example, Figure 4 shows the three lowest energy conformers of CDR-H_3_ sampled near the x-ray structure of MA2-8; there is a large conformational spread but with a relatively small energy range of 2.6 kcal/mol (well within forcefield parameterization error). This presents a problem when ranking conformations and has been observed in small molecule docking calculations.^25^ Second, forcefields are quite sensitive to small perturbations of the coordinates; experimental coordinates (which serve as the RMSD reference) are typically high in energy when compared to refined modeled structures, which, in some sense, is a built-in bias towards higher RMSD values. Given the premise of Stage 2 modeling with >90% of the heavy atom coordinates known, the energy differences among the models are caused exclusively by the conformational variability in CDR H3. Comparing the top energy models with the native x-ray structures as shown in Figure 2(B) reveals a systematic failure in discriminating the low RMSD conformer from the conformation pool. The results of a correlation analysis of AMBER12 all-atom energy against global RMSD do not support the notion of a linear relationship between the two variables [Fig. 2(B)]. This suggests that other—maybe more CDR H3-specific—terms are required to increase the loop prediction accuracy.

In retrospect, keeping all of the non-H_3_ heavy atoms fixed in our protocol may have contributed to the higher RMSD values; for example, the join residues in the *F*_V_ (being fixed) may have contributed to increased strain energy that could have been relaxed if a tethering protocol was used. Such strain would confound the ranking and perhaps the Ramachandran component of the scoring function used in CCG3 and CCG4 (and implicitly in CCG5) may help in such cases if some means were developed to combine it with the energy value.

## CONCLUSION

We have described a method for automatic modeling of the *F*_V_ region of an immunoglobulin. It is based upon the use of a precompiled antibody x-ray structure database. This database is used as a source of framework and hypervariable region structural templates, which are grafted together. Multiple initial models from multiple templates are constructed. A final consensus model is built by selecting the templates used in one or more of the initial selected by considering forcefield binding energies between *F*_V_ components along with cluster analyses of the CDRs of the initial models. In addition, we have described a method for modeling the CDR-H_3_ loop in the context of known coordinates for the remainder of an *F*_V_. The method is based on using a computationally enriched database of H_3_ conformations as a source for loop grafting and coordinate refinement.

The described methods were used in the Second Antibody Modeling Assessment (see Almagro, this issue) on commonly available desktop computer hardware; ∼30 min of CPU time were required to build each model without user intervention (except for one manually modeled rabbit *F*_V_). Stage 1 of AMA-II involved full *F*_V_ modeling and our models resulted in an average of 1.05 Å RMSD to the (previously withheld) x-ray crystal coordinates. Our previously established protocol (used in the First Antibody Modeling Assessment) proved the best when compared with two experimental new protocols still under development. Stage 2 of AMA-II focused on CDR-H_3_ modeling with known remaining *F*_V_ coordinates. Our specialized CDR-H_3_ modeling method (which was still under development at the time of the assessment) produced conformation predictions that were on average 1.5 Å local RMSD to the x-ray coordinates and 2.4 Å global RMSD. Leaving out a few problem structures resulted in a remaining average of 1.1 Å local RMSD local and 1.7 Å global.

The accuracy of CDR-H_3_ predictions (whether under Stages 1 or 2 conditions) is lower than that of the other CDR loops and the remainder of the *F*_V_ region. Our methods are capable of generating diverse numbers of CDR-H_3_ conformations and in the majority of cases considered in AMA-II there was a generated conformation <1.0 Å RMSD from the x-structure. The challenge appears to be the accurate ranking of the conformations. We present some evidence that the forcefield energies are a viable method for ranking keeping in mind that parameterization and coordinate perturbations can have a significant impact on such ranking.
